# What Place Has Low-Fusing Porcelain in Dentistry?

**Published:** 1903-06

**Authors:** Robert Brewster

**Affiliations:** Chicago, Ill.


### WHAT PLACE HAS LOW-FUSING PORCELAIN IN DENTISTRY?

BY MR. ROBERT BREWSTER, CHICAGO, ILL.

THE melting-point of fine gold is the adapted dividing line (approximately) between what is known as high- and low-fusing porcelain. Some high-fusing porcelains, however, fuse 400° or 500° above the gold melting-point, whilst some of the low-fusing bodies can only be used safely with a blow-pipe, their melting-point being so low.

It will be remembered by those who have followed the subject that the original claim for low-fusing material was that gold was more easily adapted to cavities than platina, and upon this claim its use was principally urged. There seems of late, however, and more especially since tests were made in Germany with a few of the different porcelains to determine their resistance to crushing, that less importance is placed upon that most important phase of the subject, the value of gold as a matrix, than upon the strength of the porcelain. There must be some good grounds for shifting the issue; but what are they?

It has never been claimed that any of the high-fusing porcelains in general use have shown any deficiency in respect of strength, nor that anything stronger was needed. Supposing, however, that some *one* of the low-fusing bodies was stronger than *all* the high-fusing bodies on the market (which, by the way, is not the case, as my dynamometer tests have shown). That would be but an unimportant advantage as compared with the other requirements of a first-class dental porcelain, not the least of which, among the many essentials, is translucency and life-like appearance. No one who has compared the two classes would venture to claim a superiority in this respect for the low-fusing material.

Another equally important quality is that of baking flat; that is, whilst completely filling a matrix to the margins, retain the proper contour and not ball up towards the centre.

Another very necessary quality is that of holding up sharp cusps and approximal extensions under repeated firing. How frequently it happens that judgment on the exact fulness of contour is at fault, and it becomes necessary to add further material. This is a comparatively easy task with a body which will fuse to a glaze and yet hold its form intact, but is extremely difficult with a material which, the moment it glazes, begins to change form.

A recent illustration of this decided tendency in all very low-fusing porcelains to become spheroidal, was made evident at the Odontographic Clinic held February 13, 1903, when Dr. Ottolengui (one of the most expert manipulators of the Jenkins material) challenged any Western man to burnish platina matrices and make inlays in high-fusing porcelain for the cavities he had prepared, one of which was a distal approximal in a canine, involving the whole of the cutting edge; the other, the whole grinding surface of a molar. The challenge was taken up by Dr. Reeves, of Chicago, with the result that the inlays made by Dr. Reeves with the Brewster high-fusing porcelain were pronounced by the committee of four experts to be perfect in fit and contour, whereas those made by Dr. Ottolengui with the Jenkins material did not fit.

This incident very forcibly emphasizes the difficulty experienced even by an expert, in obtaining perfect fitting inlays with material so susceptible to heat.

The subject of fusibilty was duly considered in the experiments carried out several years ago by some of the Chicago dentists and myself, when determining a formula for a reliable porcelain adapted to all classes of porcelain work, and no porcelain fusing below gold was found adequate. The result of six years' steady work, observation, and experience on the part of the profession with porcelain of the formulæ then adopted has been but to confirm the correctness of the view held at the time.

Whilst, however, the use of gold for matrices is being advocated by a few prominent men in different parts of the country, and whilst there is such a great diversity of opinion upon the subject, there will, naturally, be a desire on the part of many to investigate for themselves. As a consequence, I was asked some time since if I could not make a porcelain that would fuse upon gold and yet retain some of the characteristics of high-fusing porcelain.

Acceding to this request involved quite a long series of experiments, which, I am glad to say, have ended satisfactorily to all concerned. The departure from old lines has been radical. It would have been an easy matter to have taken my enamel body, and by treating with fluxes produced a material which, immediately upon reaching a heat sufficient to fully glaze it, would lose its form, thus requiring some special design of blow-pipe or other apparatus to handle it.

This would have been a retrograde step, not only for the important reason given, but because there would have been lessened translucency in a marked degree; in fact, I should only have produced a duplicate of the low-fusing bodies already in the hands of the profession.

The result of my experiments on entirely new lines has been to produce what is termed a "gold matrix porcelain," having all the appearances, when fused, of high-fusing material, having very considerable strength, holding up well under fire, allowing quite a margin of heat increase without change of form, an entire absence of bubbling, and a translucency not before obtained in a material fusing at so low a temperature (about the melting-point of 14-carat gold solder).

So that, whilst conceding that the bulk of opinion in this country is in favor of platina for matrices, owing doubtless, to the trend of education having been in that direction, the opportunity is now given to every one to test the question for themselves, at very nominal expense, and without any change in their present baking appliances, thus enabling them to add to the knowledge of the subject from practical experience, and be in a position to answer the question asked at the beginning of this paper.

